# CD5-Positive Intravascular Large B-Cell Lymphoma in a Patient with Wilson's Disease: Case Report and Review of the Literature

**DOI:** 10.1155/2018/5140586

**Published:** 2018-12-16

**Authors:** Neha Gupta, Chrystalle Katte Carreon, Filiz Sen, Peter Farmer, Xinmin Zhang, Silvat Sheikh-Fayyaz, Nina Haghi

**Affiliations:** ^1^Department of Pathology and Laboratory Medicine, Donald and Barbara Zucker School of Medicine at Hofstra/Northwell, 6 Ohio Drive, Suite 202, Lake Success, New York, 11042, USA; ^2^Department of Pathology, Boston Children's Hospital/Harvard Medical School, 300 Longwood Ave. Boston, MA 02115, USA; ^3^Department of Pathology, Memorial Sloan Kettering Cancer Center, 1275 York Avenue, New York, 10065, USA

## Abstract

Intravascular large B-cell lymphoma (IVLBCL) is a rare extra-nodal B-cell lymphoma that proliferates within small/intermediate blood vessels and capillaries while sparing large blood vessels and organ parenchyma. Clinical presentation is highly variable and may include B symptoms, neurological deficits, and/or cutaneous findings. The diagnosis of IVLBCL is difficult due to multiorgan involvement and nonspecific symptoms. We describe the case of a 68-year-old male who presented with progressive weakness, confusion, and falls. He had a past medical history of liver cirrhosis secondary to Wilson's disease. Physical exam and laboratory results revealed a lethargic man with jaundice, hepatic encephalopathy, and abnormal liver/kidney function tests. He expired after a short hospital course in the setting of hepatic and renal failure. Postmortem examination revealed large neoplastic lymphoid cells involving multiple organ blood vessels; however skin and neurologic involvement was absent. The neoplastic cells demonstrated B-cells positive for CD5, rendering a diagnosis of IVLBCL. Our case represents the occurrence of IVLBCL with CD5-positivity in a patient with Wilson's disease, diagnosed at autopsy demonstrating the challenging nature of diagnosing IVLBCL.

## 1. Introduction

Intravascular large B-cell lymphoma (IVLBCL) is a rare B-cell lymphoma involving and proliferating within small blood vessels and capillaries with sparing of large blood vessels [1]. Presenting symptoms are nonspecific with a majority of patients presenting with B symptoms. Common sites of involvement include vessels of the brain, bone marrow, skin, liver, and spleen [2–4].We report a case of IVLBCL presenting in a patient with clinical and laboratory features of acute hepatic encephalopathy and renal failure due to an underlying history of Wilson's disease. Diagnosis of IVLBCL was made on postmortem examination, in which multiple organ vessels were found to be involved.

## 2. Case Report

A 68-year-old male presented to the emergency department with progressive weakness, intermittent confusion, and falls. Past medical history was significant for coronary artery disease, peripheral vascular disease, hypertension, diabetes mellitus, and liver cirrhosis secondary to Wilson's disease. Physical exam revealed a lethargic but oriented patient with jaundice, superficial skin abrasions on arms, bilaterally diminished breath sounds, grade 2/6 systolic murmur, and lower extremity edema.

The patient was admitted for management of acute renal failure and hepatic encephalopathy. Complete metabolic profile revealed elevated ammonia of 186 *μ*mol/L (12-60 *μ*mol/L), serum creatinine of 6.8 mg/dL (0.7-1.2 mg/dL), blood urea nitrogen of 134 mg/dL (8-20 mg/dL), total bilirubin of 3.0 mg/dL (0.4-2.0 mg/dL), aspartate aminotransferase of 97 U/L (15-41 U/L), alkaline phosphatase of 318 U/L (38-126 U/L), and albumin of 2.4 g/dL (3.5-4.8 g/dL). Hematology and coagulation studies showed hemoglobin of 12.9 g/dL (12.5-15.5 g/dL), thrombocytopenia (platelet count of 55,000; 150,000-400,000 normal), neutrophilia (79.7%; normal range 37-73%), monocytosis (13.3%; normal range 3.0-10%), lymphocytopenia (6.3%; normal range 20-55%), high red cell distribution width of 19.7% (11.0-15.6%), and elevated activated partial thromboplastin time of 39 seconds (normal range 26-34 seconds). Serologic studies for hepatitis virus were negative. Cardiac enzymes were unremarkable. The laboratory values for lactate dehydrogenase (LDH), soluble interleukin-2 receptor, beta-2 microglobulin, and ferritin were not available. Computerized tomography scan of the head revealed no acute intracranial changes.

The patient was placed on hemodialysis and lactulose with serial monitoring of ammonia levels. His clinical status improved slightly after dialysis but showed no sustained clinical improvement. On the 6^th^ hospital day, the patient was intubated following worsening oxygenation and severe hypotension and expired the following day. A complete postmortem examination was performed.

### 2.1. Autopsy Findings

**Figure 1:**
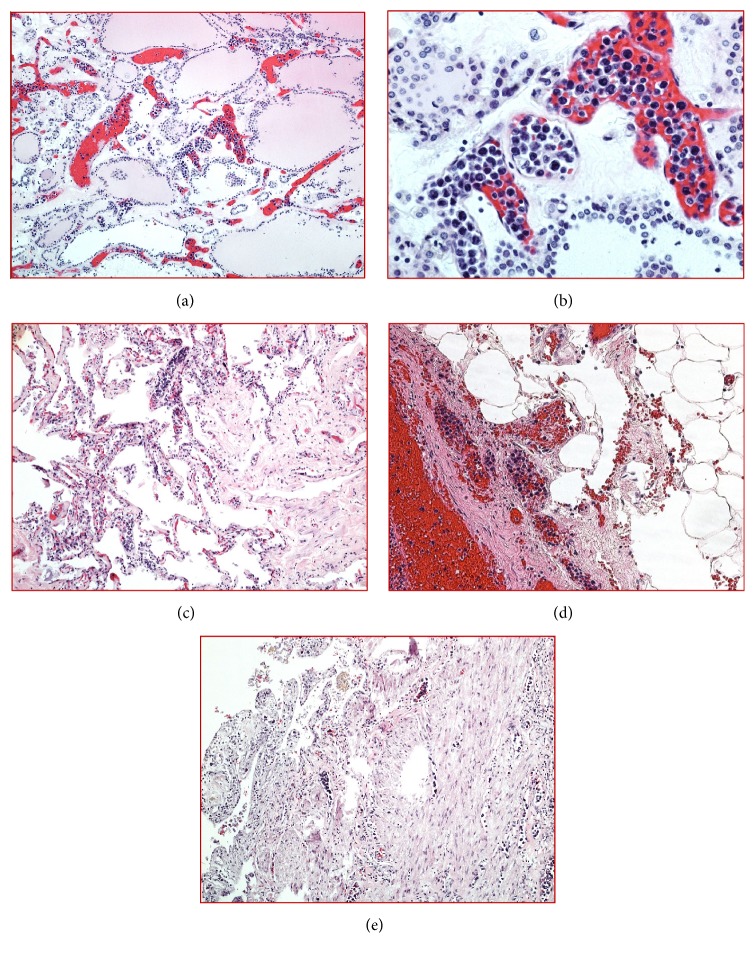
H&E. Large neoplastic lymphoid cells with hyperchromatic nuclei and coarse chromatin are present within the small blood vessels in thyroid ((a), 10X; (b), 40X), lung ((c), 10X), omentum ((d), 20X), and gall bladder ((e), 10X).

**Figure 2:**
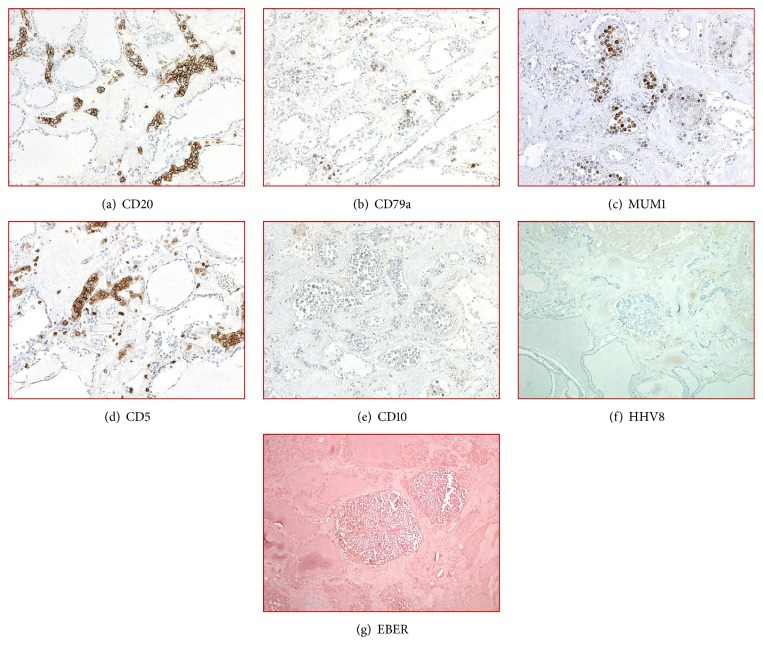
Immunohistochemical studies performed on thyroid gland revealed that the neoplastic lymphoid cells are positive for CD20 ((a), 20X), dim CD79a ((b), 20X), MUM1 ((c), 20X), and CD5 ((d), 20X); negative for CD10 ((e), 20X), HHV8 ((f), 10X), EBER ((g), 10X), c-MYC, BCL6, and cyclin D1 (not shown).

At autopsy, the patient had jaundice, bipedal edema, hepatomegaly (weight = 2219 grams) with diffuse micronodular cirrhosis, and splenomegaly (weight = 907 grams), consistent with liver failure. He had moderate cardiomegaly (weight = 533 grams) with marked concentric left ventricular hypertrophy and bilateral nephrosclerosis, consistent with long-standing hypertensive cardiovascular disease. Upon microscopic examination, numerous large atypical lymphoid cells were found within the lumen of several small blood vessels of the thyroid gland (Figures 1(a) and 1(b)), lungs (Figure 1(c)), omentum (Figure 1(d)), gallbladder (Figure 1(e)), peripancreatic tissue, and pericolonic fat. These cells were large in size, with round to somewhat irregular hyperchromatic nuclei with occasional prominent nucleoli (Figure 1(b)). Immunohistochemical staining performed on thyroid gland revealed a B-cell phenotype (positive for CD20 (Figure 2(a)), dim CD79a (Figure 2(b)), and dim PAX-5 and also positive for MUM-1 (Figure 2(c)), CD5 (Figure 2(d)), and dim CD30. CD10 (Figure 2(e)), BCL-2, c-MYC, HHV8 (Figure 2(f)), BCL-6, CD3, CD138, and cyclin-D1 were negative. Epstein Barr virus (EBV)-encoded small RNAs (EBER) by in situ hybridization was negative (Figure 2(g)). This phenotype supported nongerminal center phenotype of large B-cell lymphoma. Ki-67 showed nuclear staining in approximately 60-70% of cells. The vessels of the spleen and liver were not involved by the lymphoma. There was no involvement of the skin, bone marrow, or peripheral blood. Neuropathologic examination showed mature central nervous system with no specific histopathologic findings. Overall, the histomorphologic features and immunoprofile were consistent with IVLBCL. The clinical, laboratory, and autopsy findings demonstrated that the most likely cause of death was hepatorenal syndrome due to liver cirrhosis complicated by IVLBCL diagnosed at autopsy.

## 3. Discussion

IVLBCL is a rare type of extranodal large B-cell lymphoma first described in 1959 by Pfleger and Tappeiner [5]. It can involve any organ of the body such as bone marrow, skin, brain, lungs, liver, spleen, thyroid, and gastrointestinal tract while lymph nodes are relatively spared [1, 2, 4, 6, 7]. IVLBCL mainly affects elderly patients, with no sex predominance. Central nervous system, skin, and bone marrow are the most frequent sites of involvement [2–4]. The Western variant more often involves the CNS and skin. The Asian variant (mostly described in Japan) presents with multiorgan failure, hepatosplenomegaly, bone marrow involvement, hemophagocytosis, and more frequent CD5-positive immunophenotype [4]. Unlike its Western counterpart, CNS and skin are less commonly involved in this variant [3, 4].

Clinical manifestations are very heterogeneous and include nonspecific B symptoms while laboratory abnormalities may show anemia and elevated lactate dehydrogenase levels [2] (not known in our case). Three-quarters of patients have stage IV disease at presentation, and 21% to 34% of cases are diagnosed at autopsy (60% of which have CNS involvement) [2]. Cases have been reported in the literature in which the diagnosis of IVLBCL was made early (at presentation) with few organs involved resulting in prompt initiation of treatment with good clinical outcome [7–10]. Such cases highlight the importance of the early diagnosis in conjunction with limited extent of disease in the prognosis and management of IVLBCL. Treatments are variable and include anthracycline-based chemotherapy and high dose chemotherapy followed by autologous stem cell transplantation [2, 8, 11, 12]; however overall survival remains poor due to disseminated disease at presentation. A subgroup of patients with cutaneous-only disease (and female predominance) and early initiation of the treatment with rituximab containing chemotherapy protocols are reported to have a better overall survival [2, 4, 13]. The incidence of CNS progression during therapy or relapse is high; therefore it is suggested that specific treatments and prophylaxis for CNS recurrence be considered for all patients diagnosed with IVLBCL [12].

Our case presented with nonspecific neurological symptoms (loss of consciousness and balance difficulties), found to have acute renal failure and hepatic encephalopathy which was attributed to liver cirrhosis due to Wilson's disease. The abnormal laboratory findings of liver and kidney failure as well as deranged hematological parameters did not raise suspicion of lymphoma as these abnormalities can present in both diseases. Together with the negative brain imaging, the overall clinical findings kept clinical suspicion of any lymphomatous process very low. Detecting IVLBCL in such patients with active comorbidities is a real challenge. The antemortem diagnosis of CNS IVLBCL is especially difficult in elderly patients due to the overlapping stroke-like symptoms and nonenhancement of IVLBCL on MRI [2, 3]. In our patient, neurologic symptoms, although present, were not confirmed by histologic evidence of lymphoma involving the CNS vessels. Masaki et al. proposed an early clinical diagnostic strategy for starting chemotherapy for IVLBCL in cases in which time is a limiting factor. Included in the strategy is checking serum LDH and soluble IL2R levels (not available in our case), as these are indicators of high tumor burden. However, to start chemotherapy, pathology-confirmed lymphoma cells are required [14]. For this reason some authors suggest random skin and bone marrow smears and biopsies as helpful tools for diagnosis when suspicion is high [8, 14]. In our case, however, both skin and bone marrow proved to be uninvolved, which leaves even greater uncertainty as to how we could have confirmed presence of lymphoma cells pathologically in a timely manner. Recently, a group described the use of liquid biopsy as a useful method for diagnosing as well as monitoring IVLBCL by obtaining cell free DNA from plasma or serum and analyzing for frequent mutations of IVLBCL [15].

Histopathology is still the standard for the diagnosis of IVLBCL. Immunohistochemically, IVLBCL is positive for B-cell markers with variable aberrant expression of CD5. CD10 is found in low frequency [1]. Murase et al. reported nongerminal center phenotype as most predominant (13% CD10+, 26% BCL6+, 95% MUM1+) with 91% of cases showing BCL2 positivity [4]. CD5 positivity is found in approximately 30 to 38% of cases and is associated with frequent bone marrow and peripheral blood involvement [4, 16]. CD5 positivity, in the absence of prior history of lymphoma, is thought to be evidence of de novo IVLBCL [4, 17, 18]. Immunophenotype, however, has not been shown to affect outcomes [16]. IVLBCL has been associated with complex karyotype and structural aberrations in chromosomes 1, 6q, and 18 in over 50% of IVLBCL cases [19].

Our case is unique in that the immunophenotype showed nongerminal center phenotype with CD5 positivity (more often seen in the Asian variant); however, bone marrow and peripheral blood involvement was not found. Additionally, our patient did not have hemophagocytosis or skin/CNS involvement, thus not fitting into either Asian variant (also referred to as “IVL associated with hemophagocytic syndrome”) or Western variant [16]. To our knowledge, there is no known association described between Wilson's disease and lymphoma. Rare cases of children with Wilson's disease and acute lymphoblastic leukemia have been reported [20, 21]; however, no other cases associated with hematopoietic disorders have been described.

## 4. Conclusion

IVLBCL is an aggressive rare B-cell lymphoma with a high mortality rate. Our case demonstrates the difficulty in the diagnosis of IVLBCL, especially in the setting of neurologic symptoms and underlying liver disease due to the highly variable tissue involvement and potentially nonspecific clinical presentation. Some useful features might include neurological symptoms with negative radiological findings of a lesion or mass effect with or without skin eruptions accompanied by laboratory evidence of deranged organ function. Making an antemortem diagnosis of IVLBCL is very important given the impact of early treatment on the clinical course and prognosis of this disease. This case also illustrates the importance of careful postmortem examination and collaboration with the hematopathology team to identify and work up any atypical cells seen within the vessels, as such findings can easily go unrecognized.
